# Assessing cortical cerebral microinfarcts on iron-sensitive MRI in cerebral small vessel disease

**DOI:** 10.1177/0271678X211039609

**Published:** 2021-08-20

**Authors:** Kim Wiegertjes, Kwok-Shing Chan, Annemieke ter Telgte, Benno Gesierich, David G Norris, Catharina JM Klijn, Marco Duering, Anil M Tuladhar, José P Marques, Frank-Erik de Leeuw

**Affiliations:** 1 Department of Neurology, Donders Institute for Brain, Cognition and Behaviour, Radboud University Medical Center, Nijmegen, the Netherlands; 2 Donders Institute for Brain, Cognition and Behaviour, Centre for Cognitive Neuroimaging, Radboud University, Nijmegen, the Netherlands; 3 Institute for Stroke and Dementia Research (ISD), University Hospital LMU Munich, Munich, Germany; 4 Erwin L. Hahn Institute for Magnetic Resonance Imaging, University of Duisburg-Essen, Essen, Germany; 5 MIRA Institute for Biomedical Technology and Technical Medicine, University of Twente, Enschede, The Netherlands; 6 Medical Image Analysis Center (MIAC AG), Basel and qbig, Department of Biomedical Engineering, University of Basel, Basel, Switzerland

**Keywords:** Acute ischemia, cerebrovascular disease, cortical microinfarcts, magnetic resonance imaging, small vessel disease

## Abstract

Recent studies suggest that a subset of cortical microinfarcts may be identifiable on T2* but invisible on T1 and T2 follow-up images. We aimed to investigate whether cortical microinfarcts are associated with iron accumulation after the acute stage. The RUN DMC – InTENse study is a serial MRI study including individuals with cerebral small vessel disease (SVD). 54 Participants underwent 10 monthly 3 T MRIs, including diffusion-weighted imaging, quantitative R1 (=1/T1), R2 (=1/T2), and R2* (=1/T2*) mapping, from which MRI parameters within areas corresponding to microinfarcts and control region of interests (ROIs) were retrieved within 16 participants. Finally, we compared pre- and post-lesional values with repeated measures ANOVA and post-hoc paired *t*-tests using the mean difference between lesion and control ROI values. We observed 21 acute cortical microinfarcts in 7 of the 54 participants (median age 69 years [IQR 66–74], 63% male). R2* maps demonstrated an increase in R2* values at the moment of the last available follow-up MRI (median [IQR], 5 [5–14] weeks after infarction) relative to prelesional values (*p* = .08), indicative of iron accumulation. Our data suggest that cortical microinfarcts are associated with increased R2* values, indicative of iron accumulation, possibly due to microhemorrhages, neuroinflammation or neurodegeneration, awaiting histopathological verification.

## Introduction

Cortical microinfarcts are frequently encountered on neuropathological examination of brains of elderly individuals, especially in those with cerebrovascular disease or dementia.^1,2^ Nonetheless, only a small proportion (<1%) of the total microinfarct burden can be detected with MRI, clearly limiting our understanding of the causes and consequences of microinfarcts.^3^

In the RUN DMC – InTENse study, a monthly serial imaging study, we detected acute cortical microinfarcts (A-CMI) in 13% of the participants.^4^ However, none of the A-CMI turned into a chronically visible lesion on follow-up MRI.^5,6^ This mismatch between detection of A-CMI and chronic microinfarcts (C-CMI) may be explained by the fact that C-CMI have currently been defined to be isointense on iron-sensitive MRI sequences.^3^ A recent *in vivo* 3 T MRI study, demonstrated that 56% of the detected A-CMI demonstrated visible signal alterations on susceptibility-weighted imaging (SWI) after 1 to 3 years, whereas signal alterations on follow-up T2-weighted imaging were found in only 29% of the A-CMI.^7^

However, SWI dichotomizes tissue into abnormal and normal tissue and tends to extend regions of high susceptibility (blooming artefact) which appear as dark voids that are not reliable for quantification of the degree of iron accumulation.^8^ This limitation is overcome by using R2* mapping, which is a quantitative relaxometry metric whose changes can be linearly related to changes in local brain concentration (an increase in R2* is associated with a give increase in concentration of iron), thereby providing a detailed evaluation of the underlying tissue alterations at the voxel level.^8,9^ Therefore, the aim of this study was to investigate whether initial acute cortical microinfarcts are associated with signal alterations on R2* mapping indicative of iron accumulation after the acute stage.

## Materials and methods

### Study design and subjects

Participants were included from the RUN DMC – InTENse (Radboud University Nijmegen Diffusion tensor and Magnetic resonance imaging Cohort – Investigating The origin and EvolutioN of cerebral small vessel disease) study.^5^ Details of the study protocol and MRI processing have been described previously.^4,5^ Briefly, we selected 54 participants with progressive sporadic SVD (median [IQR] 69 years [66–74], 63% male) from the ongoing RUN DMC study. Because previous WMH progression is the most important determinant of future WMH progression, individuals were invited by volume of WMH progression on MRI scans between 2006 and 2015,^10^ while meticulously excluding those individuals with other causes of cerebral ischemia.^11^ Participants with intracranial haemorrhage on MRI (except microbleeds), dementia, Parkinson’s diseases, life expectancy <1 year, or 3 T MRI contraindication were also excluded. Participants underwent a pre-screening visit to assess study eligibility, cardiovascular risk factors and cognition and 10 monthly MRI scans.^5^ Cardiovascular risk factors were defined as previously described.^4,5^ Data were collected between March 2016 and November 2017.

### Standard protocol approvals, registrations, and patient consents

The study was approved by the medical ethics committee region Arnhem-Nijmegen. All participants provided written informed consent. The guidelines according to the Declaration of Helsinki were followed.

### MRI protocol and processing

Participants underwent monthly MRI scanning in a 3 T scanner (MAGNETOM Prisma, Siemens Healthineers, Erlangen, Germany) with a 32-channel head coil. The MRI protocol was specified in detail previously,^5^ and included the following sequences relevant to the current study: 3 D T1 mapping (MP2RAGE) for R1 mapping (=1/T1) with 0.85 mm isotropic voxels;^12^ multiple spin echo T2-weighted imaging for R2 mapping (=1/T2) and reconstructed voxel size 0.36 × 0.36 × 3.3 mm^13^; 3 D fluid-attenuated inversion recovery (FLAIR) with 0.85 mm isotropic voxels; multi-shell DWI including 90 diffusion-weighted directions (30 × b = 1000, and 60 × b = 3000 s/mm2) and 10 non-diffusion-weighted scans (b = 0) with 1.7 mm isotropic voxels and multi-band acceleration factor 3,^14^ and one b = 0 image with imaging parameters equal to the previous DWI but with reversed phase-encoding direction for susceptibility-induced distortion correction.^15^ Diffusion data were processed as described previously.^4^ Diffusion-weighted trace images were generated based on the arithmetic mean across all diffusion directions within one shell, and mean diffusivity (MD) and fractional anisotropy (FA) maps were calculated on the inner shell using dtifit in FSL;^16^ 3 D multiecho gradient echo sequence (GRE) providing magnitude and phase images (6 echoes; ΔTE = 4.92 ms) for susceptibility weighted images and R2* mapping (=1/T2*) with 0.8 × 0.8 × 2.0 mm voxels. To create the SWI images, phase images were high-pass filtered using a 2 D hamming filter with a 12 × 12 kernel across all image slices and all echoes. The high-pass filtered phase images were subsequently self-multiplied four times (i.e., m = 4), before combining to the magnitude data as described previously.^17^ The magnitude SWI images were then subsequently averaged across all echoes to produce the final SWI image.^4^ R2* maps were derived from the multi-echo magnitude GRE data, using the closed-form solution based on the trapezoidal rule to approximate the integration of the magnitude decay, as described previously,^18^ implemented in a custom-written tool (https://github.com/kschan0214/r2starmapping) in Matlab.

### Acute cortical microinfarcts

A-CMI were defined as <5 mm hyperintense lesions on DWI restricted to the cerebral cortex, with a corresponding hypo- or isointense signal on the mean diffusivity (MD) map.^4,6^ All monthly DWI scans were assessed for A-CMI and manually segmented,^4^ with high spatial overlap between segmentations of the two raters (interquartile range [IQR] = 0.8–1.0). Afterwards, the A-CMI were visually inspected on T1/FLAIR scans of the same visit as A-CMI onset and the last follow-up visit as described previously,^6^ and on SWI/R2* scans of these same visits.

### Chronic cortical microinfarcts

C-CMI were defined as lesions <5 mm in diameter restricted to the cerebral cortex, according to previously published criteria.^6,19^ Two trained raters screened all baseline and last follow-up scans, masked to clinical information.^6^ Final consensus was reached involving more raters (M.D.; S.J. van Veluw, PhD, Massachusetts General Hospital), after which true C-CMI were visually inspected on SWI/R2* scans of the first visit.

### Other MRI markers of SVD

As described previously,^4^ conventional MRI markers of SVD, including white matter hyperintensities (WMH), lacunes, and cerebral microbleeds (CMB) were assessed according to the universally standardized STandards for ReportIng Vascular changes on nEuroimaging (STRIVE) criteria.^20^ WMH volumes were reported as the proportion of total white matter volume.

### Imaging analysis

To assess lesion evolution, skull-stripped images of different modalities (T1, T2, GRE, DWI) were registered to the DWI image of the first visit using the FMRIB Software Library (FSL) Linear Image Registration Tool (FLIRT).^21^ First, images were registered to the MP2RAGE image of the same visit. This included registration between MP2RAGE and GRE images, and between MP2RAGE and DWI images. Second, registration between the different visits was performed by registering all MP2RAGE images to the one acquired in the first visit. All transformation matrices derived from the first and second steps were concatenated and applied to coregister images of the other modalities and lesion masks to the DWI image of the first visit. To minimize the effect of interscan variability, we created a control region of interest (ROI) outside the lesion by dilating the A-CMI mask one voxel in all directions followed by subtraction of the original lesion ROI. As R2* maps were subject to motion artifacts affecting different locations in the brain, which were not systematic across time points, a control ROI in the contralateral hemisphere would introduce biases in our measurements. Nevertheless, when using a control ROI in a proximal region both lesion and control ROIs should have similar biases, which could be eliminated when computing the mean difference between the two ROIs.

### Statistical analysis

Statistical analyses were performed in R (version 3.6.2; https://www.R-project.org) and α was set at .05, two-tailed. Demographics, cardiovascular risk factors and baseline SVD characteristics of participants with any (chronic or acute) CMIs were compared to those without using the Mann-Whiney test for continuous and chi-square test for categorical independent variables (or Fisher’s exact test where appropriate).

We calculated the mean of the different MRI parameters (DWI trace, MD, FA, R1, R2, and R2* values) within the lesion and control ROI at each time point. To mitigate partial volume effects from cerebrospinal fluid (CSF) and white matter, only voxels with R2* values ranging from 5 s^−1^ to 30 s^−1^ were accepted in the final analyses to avoid inclusion of voxels dominated by CSF (very low R2* values) and white matter (typically ∼30 s^−1^),^22^ while the typical cortical grey matter R2* is around 15 s^−1^ and the mean R2* of A-CMI was 19 s^−1^ across all MRI sessions in this study.^23^ The difference between the mean signal in the lesion and control ROI was used in the analysis, followed by the subtraction of the mean difference of all timepoints before lesion onset to ensure any differences between control and lesion ROIs due to residual partial volume was further minimized. To be able to study the dynamic evolution of the extracted parameters, they were temporally realigned in respect to the onset time, the visit in which the A-CMI initially appeared on the MRI (designated as “MRI 0”). We classified MRI parameters as follows: (1) MRI before the onset of a new lesion (“pre-lesional”); (2) MRI at which a new lesion is first identified (“lesional”); (3) last available follow-up MRI (“post-lesional”). We used a one-way repeated measures ANOVA and post-hoc 2-tailed paired-samples *t* tests to compare lesion values from the three time points (pre-lesional, lesional, post-lesional).

### Data availability

Data requests can be sent to the corresponding author (FrankErik.deLeeuw@radboudumc.nl).

## Results

Of the 54 included subjects (median age 69 years [IQR 66-74], 63% male) 52 underwent follow-up MRI (median total follow-up duration 40 weeks [IQR 38–40]). In total, 22 of the 54 individuals had (acute or chronic) CMIs during the study period. Individuals with any CMIs were significantly older compared to those without (median [IQR] 73 [67–80] vs 68 [65–70] years, *p* = .014, Table 1). Other baseline characteristics were similar (all *p* > .05).

**Table 1.** Group characteristics.

	No cortical microinfarcts (n = 32)	Any cortical microinfarct (n = 22)	*p*
Demographic characteristics			
Age (years)	68 (65‒70)	73 (67‒80)	**.014**
Men	17 (53%)	17 (77%)	.071
Level of education	5 (5‒6)	5 (5‒6)	.628
Cardiovascular risk factors			
Hypertension	24 (75%)	21 (96%)	.067
Diabetes	4 (13%)	2 (9%)	1.00
Hypercholesterolemia	16 (50%)	11 (50%)	1.00
BMI (kg/m^2^)	26 (4)	26 (4)	.881
Smoking (ever)	22 (69%)	16 (73%)	.753
Baseline MRI characteristics			
WMH volume^a^	0.80% (0.51–1.99)	1.54% (0.86–3.05)	.138
Lacunes, presence	5 (16%)	7 (32%)	.194
Microbleeds, presence	14 (44%)	11 (50%)	.651

^a^WMH volume was calculated as (WMH volume/WM volume) × 100.

**Figure 1.**
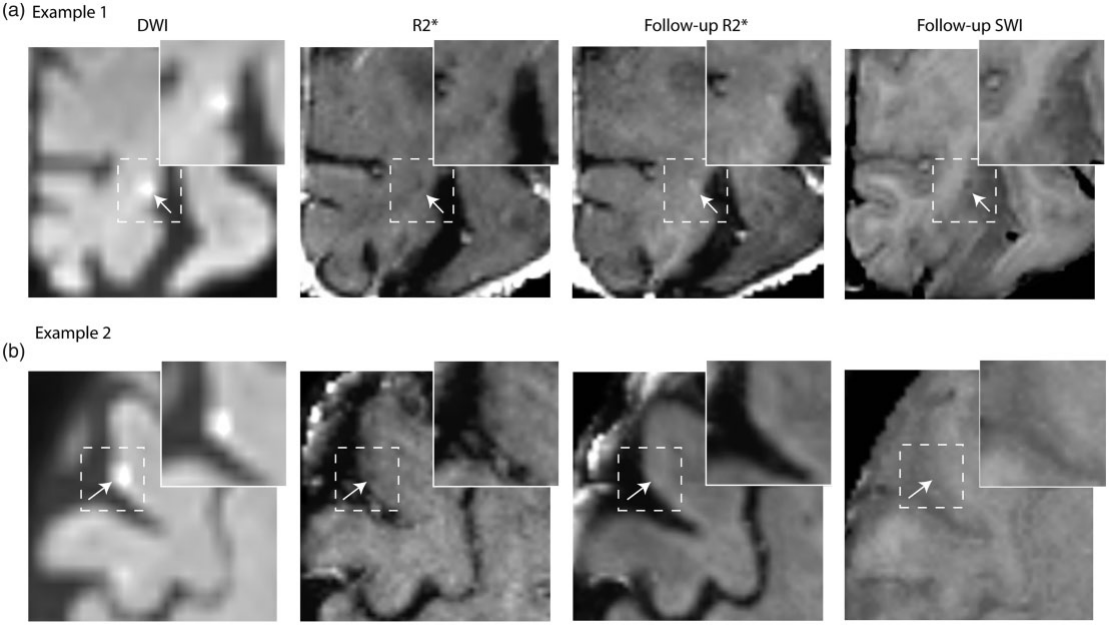
Examples of R2* signal change at the location of acute cortical microinfarcts. Acute cortical microinfarcts detected on diffusion-weighted imaging (DWI) trace images (shown here b = 3000), accompanied by a hypointense signal on R2* in the acute stage in both panel A and in panel B. On the last available follow-up MRI, the microinfarct in panel A is characterized by an increased signal on R2* and a decreased signal on SWI, indicative of iron accumulation, whereas in panel B no visible signal alterations were observed on either R2* or SWI images at the location of the microinfarct (B).

Between March 2016 and November 2017, we observed 21 A-CMI in 7 of the 54 participants (13% [0.06–0.24]).^6^ Of these, 20 (95%) were visible on FLAIR and T1 images at the moment of appearance, 9 (43%) were hypointense on R2* maps in the acute stage (Figure 1). Of these 9 A-CMI, two were hyperintense and seven were isointense on SWI images. The remaining 12 A-CMI (57%) were isointense on both R2* and SWI. For 20 of the 21 A-CMI (95%), one or more follow-up MRI scans were available. Whereas none of the A-CMI was visible on the FLAIR and T1 images of the last available follow-up MRI, 4/20 (20%) A-CMI demonstrated a hyperintense signal on R2* maps and a hypointense signal on SWI images, indicative of iron accumulation (Figure 1). These 4 A-CMI all demonstrated a hypointense R2* signal at the moment of appearance. None of the other 16 A-CMI were visible on follow-up MRI (R2* or SWI images).

**Figure 2.**
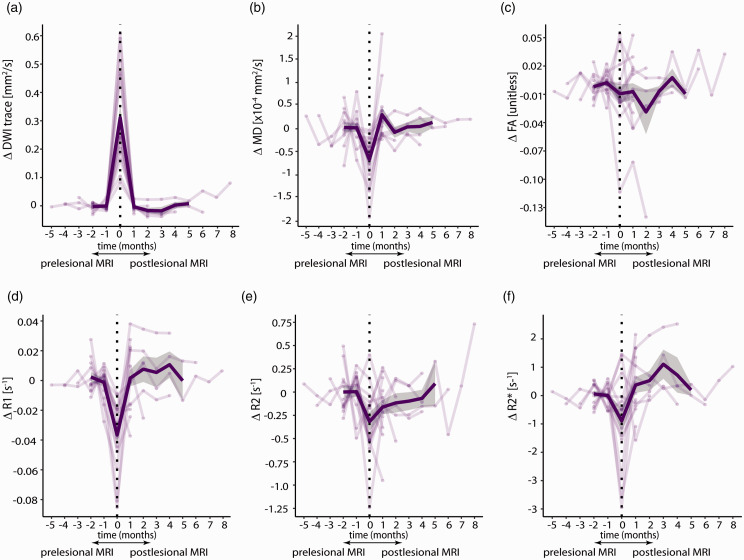
Longitudinal changes in MRI parameters at the location of acute cortical microinfarcts. Different MRI parameters are shown: (A) diffusion-weighted imaging trace (DWI), (B) mean diffusivity (MD), (C) fractional anisotropy (FA), (D) longitudinal relaxation rate (R1), (E) transverse relaxation rate (R2), and (F) Apparent transverse relaxation rate (R2*). Plotted transparent lines correspond to individual lesions and indicate the normalized mean difference between the lesion and control ROI at each time point. The mean and the standard deviation (SD) are highlighted as solid lesions and shaded regions.

For, 15 of the 21 (71%) A-CMI time series data analysis could be performed. We excluded four A-CMI because they appeared on the first or last available follow-up MRI and therefore either pre- or post-lesional values were unavailable, and two because they were located too close to the pial surface to exclude partial volume effects. There was a statistically significant main effect of time on DWI trace values (p < 0.001). At the moment of occurrence, DWI trace values were significantly increased compared to pre-lesional values five weeks earlier (median [IQR], 5 [4–5]) and post-lesional values (both *p*<.001; Figure 2) five weeks later (median, [IQR], 5 [5–14]). However, post-lesional values were similar to pre-lesional values. Moreover, we found a significant effect of time on MD values (p < 0.001). Specifically, MD was significantly lower compared to its pre-lesional value at the time of lesion appearance (*p* = .002). Post-lesional MD values were significantly higher than lesional values (p < 0.001), whereas values of the last available follow-up MRI were not statistically different from pre-lesional values. There were no significant changes in FA values over time at the location of the lesion. However, there was a statistically significant main effect of time on R1 values (p < 0.001). At the time of the lesion detection, R1 values were significantly lower than pre-lesional (p < 0.001) or post-lesional values (p = 0.002). At the last available follow-up MRI, values returned to pre-lesional R1 values. R2 values demonstrated a significant change over time (p = 0.036). At the time of lesion detection, R2 demonstrated a significant decrease compared to pre-lesional values (*p* = .019), whereas lesional values were similar to values of the last available follow-up MRI. Moreover, post-lesional did not differ significantly from pre-lesional values. Lastly, R2* values demonstrated a significant change over time (p = .017). A negative trend was observed for R2* values, with R2* values decreasing from pre-lesional levels at the moment of A-CMI appearance (p = .066). Compared to lesion values, R2* values significantly increased at the moment of post-lesional follow-up MRI (p = .015). Thereafter, R2* values showed a non-significant increase to higher than pre-lesional levels at the moment of the post-lesional follow-up MRI (*p* = .077). Furthermore, these observations were unchanged when choosing other control regions further away from the cortical infarct.

**Figure 3.**
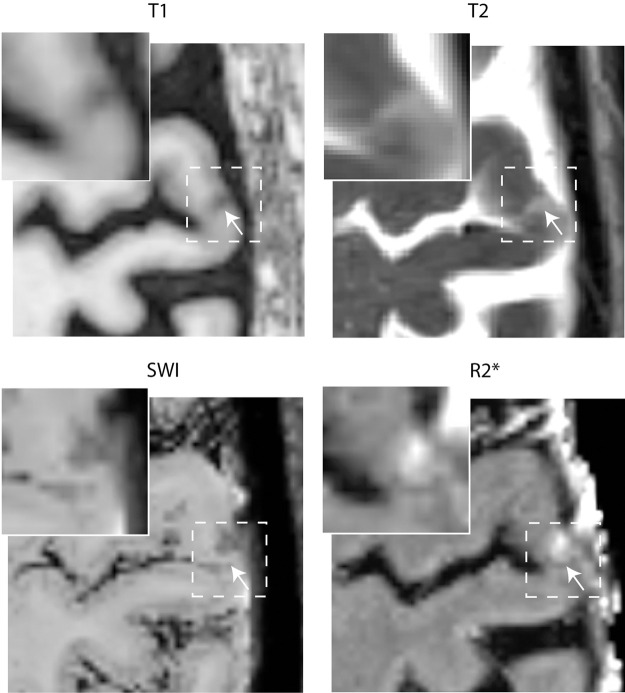
Example of R2* signal change at the location of a chronic cortical microinfarct. Example of a chronic cortical microinfarct demonstrating iron accumulation, characterized by a hypointense signal on T1, a hyperintense signal on T2, a hypointense signal on susceptibility-weighted imaging (SWI), and a hyperintense signal on R2*.

To investigate the prevalence of iron-positive microinfarcts in the chronic stage, we assessed the R2* signal change in the 81 C-CMI detected previously in 19 of the 53 participants (35% [95% CI 0.24–0.49]) of the RUN DMC – InTENse study population.^6^ Of these, 17 (21%) were hyperintense on R2* maps and hypointense on SWI images on the baseline MRI (Figure 3), consistent with the accumulation of iron. Whereas, 38 of the 81 (47%) C-CMI were hypointense on baseline R2* maps demonstrating CSF-like properties (35 showed cavitation on FLAIR images). These 38 C-CMI were all isointense on SWI baseline images. The remaining 26 C-CMI were isointense on both R2* maps and SWI images of the first visit.

## Discussion

We found an increase in R2* at the location of A-CMI, suggestive of iron accumulation, whereas the changes found in diffusion, R1 and R2 parameters at the moment of appearance of A-CMI disappeared from follow-up MRI. These results suggest that we should reconsider the formerly established criteria for the rating of microinfarcts,^3^ as C-CMI have currently been defined to be isointense on iron-sensitive MRI scans.

Traditionally, MR lesions visible on GRE are believed to represent accumulation of iron consistent with hemosiderin-laden macrophages in perivascular tissue, corresponding to vascular leakage of blood cells.^24–27^ Inherently, this suggests that the observed iron accumulation as picked up by R2* at the site of A-CMI could be the result of hemorrhagic transformation. This is in line with recent *in vivo* studies that described hemorrhagic transformation of subcortical DWI+ lesions into a MRI-detectable microbleed,^4,28^ and with the small number of post-mortem MRI studies that have demonstrated a shared underlying histopathology of cerebral microbleeds and microinfarcts.^29,30^ Furthermore, a previous neuropathological study demonstrated different types of C-CMI using 7 T post-mortem MRI, including C-CMI with or without cavitation, and C-CMI with or without hemorrhagic components.^30^

However, the more subtle changes in iron (as picked up by the quantitative R2*) could have more potential underlying causes. A combined *in vivo* histopathology-MRI study found several pathological changes at the core of C-CMI, including extracellular accumulation of lipofuscin and depletion of neurons, and activated macrophages and astroglia with a varying degree of iron accumulation.^31^ These changes were not found at the location of A-CMI. First, the extracellular accumulation of lipofuscin could be one possible source of iron. Lipofuscin aggregates as a result of neuronal cell death, and could therefore signify the beginning of tissue necrosis, a process which could ultimately result in C-CMI with cavitation.^32,33^ Another explanation could be the activation of macrophages associated with neuroinflammation,^34^ characterized by the presence of microglial cells which have been described to have a high content of ferritin.

The changes found in R1 and R2 parameters at the moment of A-CMI appearance showed normalization already on the subsequent follow-up MRI, consistent with our previous findings.^6^ However, because we were not able to investigate the disappearing A-CMI on brain pathology, it remains unclear whether this indicates tissue salvation or whether tissue damage persists that is not visible on *in vivo* 3 T MRI. Prior research on subcortical DWI lesions has demonstrated that of the DWI+ lesions that cavitated on follow-up MRI, in general only a small part of the initial DWI+ lesion showed cavitation, whereas the largest part of the DWI+ lesion had no signal abnormality on follow-up MRI.^35^ As CMI are typically very small in size (between 0.05 and 5 mm in diameter), the proportion of the tissue that remains visible on follow-up MRI might be too small to be detected with *in vivo* 3 T MRI. Although we are unsure whether these findings on subcortical DWI+ lesions can be translated to the cortex, several MRI studies demonstrated that especially small cortical infarcts seem to disappear during follow-up.^4,36,37^

Our findings accentuate the notion that cortical microinfarcts affect brain structure beyond the acute period. Due to the poor spatial and temporal resolution of *in vivo* MRI, a single A-CMI large enough to be detected on DWI scans, although having a limited effect by itself, may be indicative of a yearly incidence of hundreds of new microinfarcts,^38^ which all together may have a substantial effect on the brain. Furthermore, it has recently been suggested that microinfarcts could be associated with focal cortical atrophy,^3,37,39^ in which accumulation of iron could be a key mechanism. Future *in vivo* combined histopathology-MRI studies should investigate the causes and consequences of iron accumulation in the evolution of A-CMI into C-CMI, and its effect on the surrounding brain structure.

This study has several strengths and limitations. This is, to the best of our knowledge the first prospective *in vivo* study which investigated the R2*-based quantification of iron deposition, in the evolution of A-CMI to C-CMI. Major strengths include its monthly imaging and the use of quantitative high-resolution MRI data. Furthermore, our participants represent a well-defined SVD sample. However, due to the low sample size and small number of A-CMI, this study might have been underpowered to find associations with small or medium effect sizes. Therefore, a future study with a larger sample size could be beneficial to investigate lesion development using quantitative MRI. Second, we cannot exclude errors due to registration processes or partial volume effects, as microinfarcts are by definition located on the cortical surface of the brain, thus very close to tissue boundaries. However, we assume these errors to be small as all images were thoroughly checked using visual inspection. Third, the spatial resolution of the R2* data might have limited our analysis. Especially, small DWI+ lesions, significantly below the 2.0 mm slice thickness, might have gone undetected because of partial volume effects, and consequently R2* changes at visual assessment might have been missed. Future studies should acquire R2* data with higher through-slice resolution to limit the intra-voxel partial volume effect and with longer echo times to ensure higher SNR of the R2* maps. Fourth, we were not able to pathologically confirm that the observed increases in R2* indeed represent iron accumulation. While previous studies using a combined MRI-histopathology approach demonstrated a high correlation between R2* values and iron concentration in grey matter,^22,40^ other local background sources confound the measurement of tissue iron content as they also relate to the R2* signal, such as cell density or other trace elements.^9^ Furthermore, the observed changes in magnetic susceptibility could be due to other causes than iron depositions. For instance, deoxygenated hemoglobin trapped in small blood clots or in venous blood could also result in fast R2* changes. Nevertheless, the presence of blood clots in blood vessels of otherwise healthy and asymptomatic individuals seems rather unlikely. Moreover, both an increase in myelination and an increase of iron concentration could result in an increased R2* signal. As a result, if iron deposition occurs concurrently to demyelination, R2* changes would be smaller than expected and underestimate the increase of iron deposition. However, since we focussed on cortical grey matter regions in this study, a region of low myelin concentration (when compared to the white matter), the contribution of myelin to the R2* signal is less significant.

These findings indicate that some cortical microinfarcts detected *in vivo* are characterized by iron accumulation, possibly due to microhemorrhages, neuroinflammation, or neurodegeneration, awaiting further histopathological verification. This may call for inclusion of iron-sensitive MRI scans in the MRI requirements for the detection of CMI. Future *in vivo* combined-histopathology-MRI studies should further investigate the causes and consequences of iron accumulation in the evolution of A-CMI into C-CMI.
